# Influence Factors for Decision-Making Performance of Suicide Attempters and Suicide Ideators: The Roles of Somatic Markers and Explicit Knowledge

**DOI:** 10.3389/fpsyg.2021.693879

**Published:** 2021-09-14

**Authors:** Lingling Wang, Jingmin Li, Hailing Liu, Zhongpeng Wang, Li Yang, Li An

**Affiliations:** ^1^School of Education, Tianjin University, Tianjin, China; ^2^Institute of Applied Psychology, Tianjin University, Tianjin, China; ^3^Faculty of Psychology, Tianjin Normal University, Tianjin, China; ^4^Tianjin Vocational Institute, Tianjin, China; ^5^Tianjin University of Technology, Tianjin, China; ^6^School of Precision Instrument and Opto-Electronics Engineering, Tianjin University, Tianjin, China

**Keywords:** decision-making, suicide attempter, suicide ideator, somatic marker, explicit knowledge

## Abstract

Impaired decision-making has been observed in suicide attempters during the Iowa Gambling Task (IGT). Decision-making performance is influenced by somatic markers and explicit knowledge, but it is still unclear of the influencing role on decision-making performance in suicidal individuals. We aimed to investigate whether there is a decision-making deficit in suicide attempters, suicide ideators, as well as the distinct roles of somatic markers and explicit knowledge wherein. Thirteen suicide attempters, 23 suicide ideators, and 19 healthy controls performed the IGT. Both somatic markers (by the skin conductance responses, SCRs) and explicit knowledge (by the subjective experience rating and a list of questions) were recorded. No significant differences were found among the three groups on IGT performance, explicit knowledge, and anticipatory SCRs. IGT Performance of suicide attempters was positively correlated with explicit knowledge index while behavior performance was positively associated with the SCRs in healthy controls. These results indicate that the suicide attempters seem to apply a compensatory strategy by mostly utilizing explicit knowledge to perform normally as healthy controls in the IGT.

## Introduction

Suicide is a serious public health issue. It is the second leading cause of death among 15–29-year-olds (World Health Organization, 2017). According to the stress-diathesis model of suicidal behavior, suicide is regarded as the result of an interaction between environmental stressors and trait-like diatheses or susceptibilities to suicidal behavior, independent of psychiatric disorders (Mann et al., 1999; van Heeringen and Mann, 2014; Mann and Rizk, 2020). In recent years, many researchers have focused on the underlying neuropsychological and neurobiological mechanisms of suicide to better understand the behavior and predict who is at risk and who is not (Desmyter et al., 2011; Falcone et al., 2018). In addition, a large number of studies have revealed neurocognitive deficits in suicide attempters (Jollant et al., 2011; Richard-Devantoy et al., 2013; Giner et al., 2016). As suicide may be considered as an outcome of an altered decision, decision making deficit may be a causal cognitive factor in suicidal behaviours (Dombrovski et al., 2010).

Jollant et al. (2005) first used the Iowa Gambling Task (IGT) to explore the performance of decision-making in affective suicidal patients and found that violent suicide attempters performed significantly worse than affective control subjects indicating the possible relationship between impaired decision-making and suicide. In a large comorbid psychiatric population, it has been found that the history of suicide attempts was significantly and independently associated with impaired decision-making (Jollant et al., 2007). Moreover, it seems to be reliable and stable that the decision-making impairment is associated with the vulnerability to suicidal behavior because it has been replicated in adolescents (Bridge et al., 2012; Ackerman et al., 2015), old-aged (Clark et al., 2011), wide-range aged (from youth to old age) suicide attempters with affective diagnosis (Jollant et al., 2005, 2007, 2010), and in those from non-clinical samples (Chamberlain et al., 2013). The meta-analysis notably confirmed a significant association between disadvantageous decision-making and suicidal behavior especially with violent means revealed that decision-making deficit may be an important factor of suicide vulnerability (Richard-Devantoy et al., 2014; Perrain et al., 2021). However, the number of studies to explore the decision-making performance of suicide ideators was rare, and the results were inconsistent (Westheide et al., 2008; Sheftall et al., 2015).

Many studies have explored the influencing factors of decision-making performance. The somatic marker hypothesis proposes that emotions play an important role in decision-making and emotion-related signals (somatic markers) measured by the skin conductance responses (SCRs), which are necessary to guide choices in an advantageous direction, especially under conditions of uncertainty (Bechara et al., 1997; Bechara and Damasio, 2005). Anticipatory SCRs for bad decks were higher compared to good decks during the IGT (Wagar and Dixon, 2006). In the article, Bechara et al. (1997) reported that overt reasoning on declarative knowledge was required for advantageous decisions, and normal subjects had consciously available knowledge to guide their decision-making. Verbal reports also reflect explicit knowledge that would instruct their decision-making performance when people behave advantageously (Maia and McClelland, 2004). Some researchers even found that only explicit knowledge was sufficient to guide IGT behaviors before differential somatic activity, and the somatic markers were not critical to succeed in the IGT (Gutbrod et al., 2006; Fernie and Tunney, 2013). The level of explicit knowledge gradually improved through the IGT, and both explicit knowledge and somatic markers are shown to be involved in decision-making in healthy subjects, which implicated that advantageous decision-making seems to be associated with two systems, namely, implicit and explicit systems (Guillaume et al., 2009). It has been found that suicide attempters exhibited the decision-making impairment with a disconnection between what they “know” and what they “do”, i.e., suicidal people could not make the correct choices even if they had some level of explicit knowledge (Jollant et al., 2013). Besides, it has been found that the decision-making impairment of suicide attempters was correlated with affective lability measured as the trait, which may provide some piece of evidence for the somatic marker hypothesis in suicidal context (Jollant et al., 2005, 2010). To date, no studies have investigated the influence of both implicit and explicit systems on decision-making performance in suicide.

In the current study, we aimed to examine whether there was a decision-making deficit in non-clinical college students with suicidal thoughts or suicide attempts. We assessed both somatic markers (by the SCRs) and explicit knowledge (by the subjective experience rating and a list of questions) to explore the roles of implicit and explicit systems in the decision-making performance of suicide. Suicide attempters were hypothesized to perform worse than healthy controls and suicide ideators in a decision-making task and both explicit (explicit knowledge) and implicit systems (somatic markers) contributed to the decision-making deficit of suicide attempters (Hypothesis 1). Furthermore, no significant difference in decision-making performance was found between suicide ideators and healthy controls (Hypothesis 2).

## Materials and Methods

### Subjects and Experimental Design

Participants comprised college students aged 16–24 years, recruited from a university in Tianjin, China, as a part of a large questionnaire study exploring the influencing factors of suicide. According to the characteristics of suicide, they were divided into suicide attempt (SA) group (*n* = 13), suicide ideation (SI) group (*n* = 23), and healthy control (HC) group (*n* = 19). According to the Colombia Suicide Assessment Classification (C-CASA) and the Colombia Suicide Severity Rating Scale (C-SSRS) (Posner et al., 2007, 2011), *suicide ideation* was defined as passive thoughts about wanting to be dead or active thoughts about killing oneself, not accompanied by preparatory behavior. A *suicide attempt* was defined as potentially self-injurious behavior, associated with at least some intent to die, as a result of the act, including an interrupted attempt and aborted suicide (Posner et al., 2007). All participants were interviewed by an experienced psychiatrist with the Mini International Neuropsychiatric Inventory (MINI 5.00) to confirm the psychiatric diagnosis and suicidal history. The control subjects had no personal suicidal history and any psychiatric diagnosis. This study was approved by the ethical committee of Tianjin University, and all participants signed informed consent before the experiments.

### Assessments

#### The IGT

We tested decision-making performance using the computerized version of IGT (Overman and Pierce, 2013). It consists of four decks of cards, each labeled as decks A, B, C, and D (Bechara et al., 1994, 1999). Turning any card from deck A or deck B yields 100, and turning any card from deck C or deck D yields 50. However, some cards also carry penalties, generating a large loss of 1,250 for every 10 cards of decks A and B and a small loss of 250 for every 10 cards for decks C and D. Therefore, decks A and B are risky (bad) cards with large immediate gains but long-term losses. In contrast, decks C and D are safe (good) cards with small immediate gains, but a net gain in the long run.

Participants were with a loan of 2,000 facsimile renminbi (RMB) at the beginning and were asked to win as much money as possible. They are not told how many card selections must be made (the task is stopped after a series of 200 card selections). The net score of each subject is calculated as the difference between the number of safe and risky choices, i.e., net score = (C + D) – (A + B), for the 200 choices (total score). The scores of five blocks consisting of 40 choices are also calculated for each subject, indicating changes in the pattern of choices during the game. Positive net and block scores indicate advantageous decision-making.

Instructions on the screen during the choice period were “please consider which cards to choose”, and the time was fixed to 5,000 ms. After the end of the time, the guide was “please choose”, so the subjects can choose one card by clicking the mouse. Outcomes were given as “you win $X” or “you lose $Y”.

#### Experimental Instrument

The skin electric response was measured by the MP150WS system (BIOPAC System, Inc.) at a rate of 1,000 samples per second. With two computers, one is equipped with E-Prime software to control and present experimental materials, and the other is equipped with Acqknowledge 4.3 (HongKong HTR Co., Limited, China) to record and collect data. Different keystrokes will be marked with different markers. During the whole experiment, two computers realized data communication through COM port (HongKong HTR Co., Limited).

#### SCRs Recording

Electrodes were attached to the distal phalange of the first and second digits of the non-dominant hand. SCRs were recorded continuously throughout the task. Anticipatory SCRs were defined as SCRs generated during the period of the 5,000 ms interval of the selection of a deck. We analyzed the median maximal anticipatory amplitudes before the advantageous or disadvantageous choices using Matlab R2011b software. We introduced a variable, named autonomic response, defined as the median maximal anticipatory SCRs for the disadvantageous decks (A and B) minus the median maximal anticipatory SCRs scores for the advantageous decks (C and D), i.e., the difference between anticipatory SCRs before advantageous and disadvantageous choices (Guillaume et al., 2009).

#### Assessment of Explicit Knowledge

The explicit knowledge was assessed by the subjective experience rating and general conscious knowledge. After every block of 40 card selection, the participants were asked to provide subjective ratings about each deck of cards, in terms of how “good” or “bad” they felt each deck was on a 1–9 Likert-type scale. The specific instructions were “So far, according to your choice, I would like you to give each deck of cards a score, based on how good or bad you feel they are. That is, one indicates that you think the deck is very poor, and nine indicates that you think the deck is very good”. The questions and choices were presented on screen, and participants typed their responses to each of the questions. In addition, the subjective experience scores were analyzed by subtracting ratings of bad decks from ratings of good decks (Bowman et al., 2005).

At the end of the game, each participant was asked a list of questions. The questions were (1) tell me all you know about this game; (2) did you find any difference between the decks?; (3) suppose you select 10 new cards from the deck A/B/C/D, will you on average win or lose money? (The question is repeated for each deck.); and (4) retrospectively, if you have to choose only one deck, which one will you choose to earn as much money as possible? (Maia and McClelland, 2004; Guillaume et al., 2009). According to the answers, we assessed the level of general conscious knowledge of subjects, which was carried out as described by Maia and McClelland (2004). There are three levels of conscious knowledge: (1) level 0: the participants do not have any conscious knowledge specifying a preference for one of the two best decks; (2) level 1: the participant has conscious knowledge specifying a preference for one of the two best decks but does not have conscious knowledge about the outcomes of the decks that could provide a basis for that preference; and (3) level 2: the participant has conscious knowledge specifying a preference for one of the two best decks and has conscious knowledge about the outcomes of the decks that could provide a basis for that preference.

#### Psychometric Measures

We used the Beck Scale for Suicide Ideation-Chinese Version (BSI-CV) to the assessment of suicidal ideation (Li et al., 2010b). The Beck Depression Inventory (BDI) and State Anxiety Inventory (S-AI) were used to evaluate the levels of depression and anxiety (Wang, 1999). Personality traits were assessed by the Difficulties in Emotion Regulation Scale (DERS) and Trait Anxiety Inventory (T-AI) (Wang et al., 2007).

### Statistical Analysis

Statistical analyses were conducted with SPSS 20.0 (IBM SPSS Statistics). The characteristics of the sample were described using mean and SD for quantitative variables and proportions for categorical variables. The general conscious knowledge and gender distribution differences were compared by using Chi-square tests. The ANOVA was conducted to test for group differences in IGT net scores, subjective experience, anticipatory SCRs, psychological variables, and other demographic continuous variables. The Bonferroni *post-hoc* comparison test was used when significant the main effects were present. Pearson's correlation coefficients were calculated to test for the associations between clinical variables and IGT performance. All statistical tests were two-tailed, and *p* < 0.05 were considered statistically significant.

## Results

### General Variables

Clinical, demographic, and personality characteristics of subjects are shown in Table 1. No differences were found between groups in terms of gender, depression, state anxiety, and personality traits. There were significant differences in age between SA and HC groups (*p* < 0.05). SA and SI groups had significantly higher scores of suicide ideation compared with the HC group (*p* < 0.01; *p* < 0.05), with no difference between the two suicidal groups (*p* > 0.05).

**Table 1:** Demographic variables, clinical variables, personality variables, and MINI diagnosis among three groups.

	**SA group (** ***n*** **= 13)**		**SI group (** ***n*** **= 23)**		**HC group (** ***n*** **= 19)**		***F***	***p***
	***M***	***SD***	***M***	***SD***	***M***	***SD***		
Age (years)	18.84	1.14	20.13	2.20	20.84	2.01	4.12	0.02^*^
Depression	9.92	8.63	8.91	7.29	5.37	4.73	2.07	0.13
Suicide ideation	16.08	7.11	11.84	9.38	4.28	1.90	11.73	0.00^**^
State anxiety	36.69	7.31	35.65	7.32	35.79	8.00	0.09	0.92
Trait anxiety	42.92	6.36	43.87	9.08	42.74	6.82	0.13	0.88
**Dysregulation:**								
Emotional perception	12.69	3.53	13.48	4.29	15.79	3.84	2.82	0.07
Emotional acceptance	11.77	4.04	11.77	5.27	12.32	4.00	0.08	0.92
Emotional understanding	10.92	2.66	10.13	4.10	10.12	1.91	0.32	0.73
Target behavior	14.00	5.49	14.74	4.85	12.42	3.59	1.33	0.27
Impulse control	11.77	4.27	12.43	5.71	10.47	2.80	0.98	0.38
Strategy use	16.64	6.35	18.57	7.92	16.74	4.19	0.56	0.58
	***n***	***%***	***N***	***%***	***n***	***%***	***χ*** ^**2**^	***P***
Sex (male)	6	46.15	16	69.57	9	47.37	2.81	0.25
**MINI diagnosis**								
Major depressive episode	1	7.69	-	-	-	-		
Social phobia	3	23.08	-	-	-	-		
Generalized anxiety disorder	1	7.69	-	-	-	-		

** *p < 0.01*.

* *p < 0.05*.

### IGT Performance

There was no significant difference among the three groups on the total net score in the IGT (*p* > 0.05), and the result was unchanged after controlling the age (*p* > 0.05; Table 2).

**Table 2:** Overall performance of different groups of subjects on the IGT.

	***n***	***M***	***SD***	***F***	***p***
SA group	13	26.08	56.36	0.02	0.98
SI group	23	30.00	69.73		
HC group	19	29.57	61.03		

**Figure 1:**
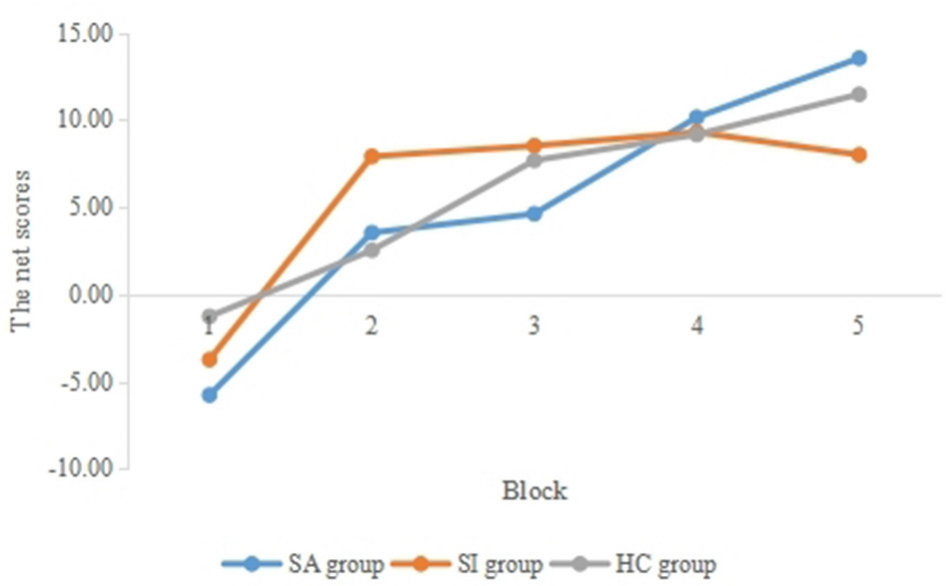
Average of net scores in five blocks of different groups.

We used 3 (group) × 5 (block) repeated measures ANOVA to explore the difference in decision-making performance in three groups over time. The results showed that the main effect of the block was significant (*F* = 9.75, *p* < 0.01, $\eta_{p}^{2}$ = 0.16), with a continuous increase in IGT net scores from block 1 to block 5 (see Figure 1). The interaction effect between group and block was not significant (*p* > 0.05), and the main effect of the group was not significant (*p* > 0.05). After controlling the age as the covariate, the main effect of the block was no longer significant (*p* > 0.05).

### Conscious Knowledge

#### Subjective Experience

**Figure 2:**
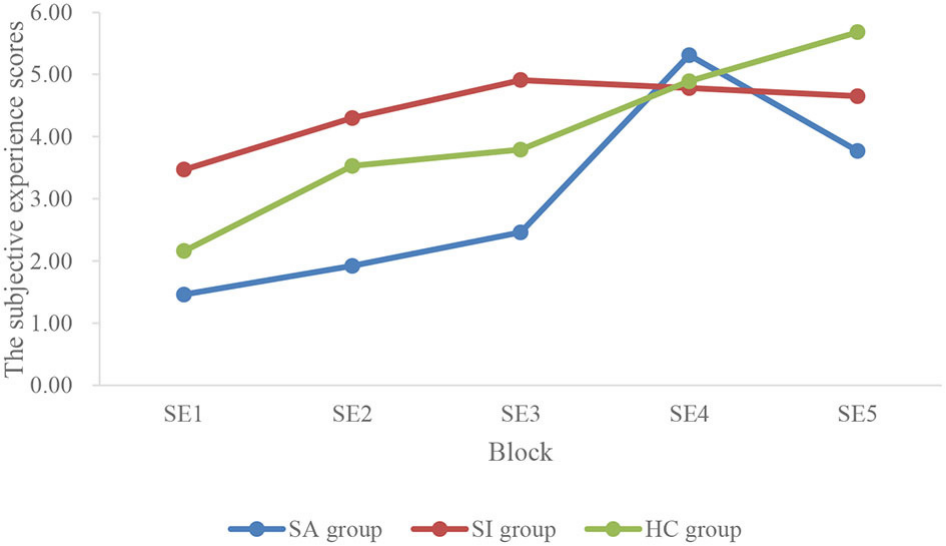
Average subjective experience scores in five blocks of different groups.

The subjective experience scores of three groups were analyzed with 3 (group) × 5 (block) repeated measurement ANOVA. The main effect of the block was significant (*F* = 7.23, *p* < 0.01, $\eta_{p}^{2}$ = 0.12), which showed that the score of subjective experience was higher as time goes on (see Figure 2). The main effect of the group was not significant (*p* > 0.05), and the interaction effect between group and module was not significant (*p* > 0.05). Finally, the main effect of the block was no longer significant when taking age as the covariate.

The relationships between subjective experience and net scores of five blocks are shown in Table 3 in the whole subjects. There were significant correlations between IGT net scores of five blocks and the subjective experience scores.

**Table 3:** Correlations between subjective experience scores and IGT net scores of five blocks in the whole subjects.

	**SE1**	**SE2**	**SE3**	**SE4**	**SE5**
Block 1	0.38^**^	–	–	–	–
Block 2	0.41^**^	0.51^**^	–	–	–
Block 3	0.10	0.38^**^	0.47^**^	–	–
Block 4	0.18	0.28^*^	0.49^**^	0.39^**^	–
Block 5	0.14	0.22	0.36^**^	0.39^**^	0.40^**^

** *p < 0.01*.

* *p < 0.05*.

#### General Conscious Knowledge

To improve the power of the statistical test, the subjects with levels 0 and 1 were integrated into one group. Therefore, we tested the net score differences between 0–1 and 2 levels of overall explicit understanding. The Chi-square test showed that there was no significant difference among the groups at different levels of conscious knowledge (*p* > 0.05; Table 4).

**Table 4:** Distribution of subjects in overall explicit understanding level.

	**Levels 0 and 1**	**Level 2**	***χ*** ^**2**^	***p***
SA group	6	6	3.20	0.20
SI group	6	17		
HC group	4	15		

*One subject in the SA group had an absence of explicit understanding data*.

We analyzed the relationship between the levels of general explicit understanding and net scores of the IGT in all participants. The results of *t*-test showed that the difference between the IGT net scores of subjects with level 2 (*n* = 38, *M* = 38.32, *SD* = 56.39) and those of subjects with level 0–1 (*n* = 16, *M* = 6.56, *SD* = 69.34) was close to significant (*p* = 0.08). There was no significant difference in IGT net scores between different levels of general explicit understanding in any of the three groups (*p* > 0.05).

**Figure 3:**
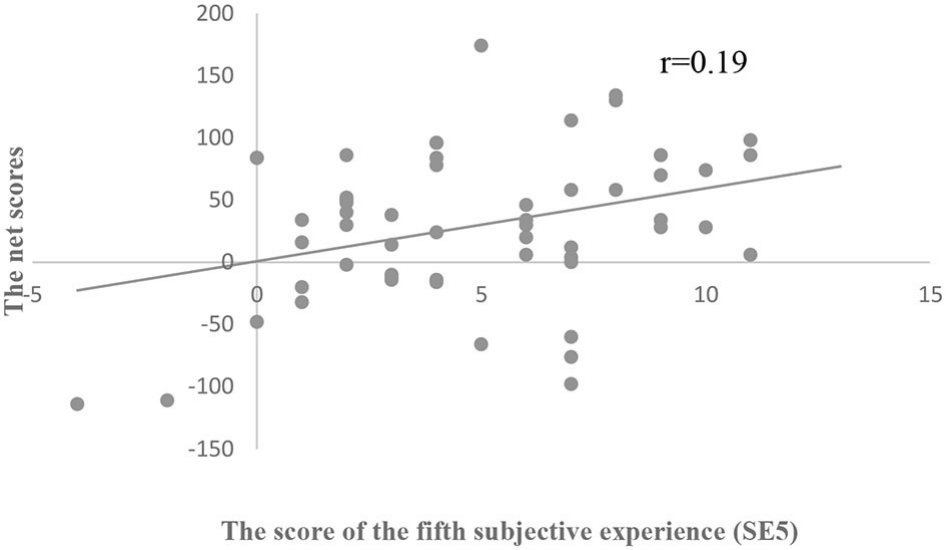
Scatterplot of the relationship between IGT net scores and SE5 among all participants.

**Figure 4:**
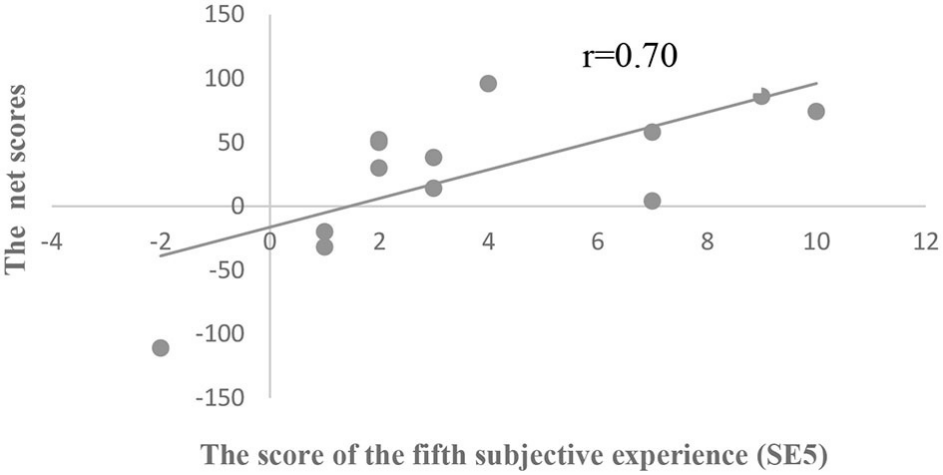
Scatterplot of the relationship between IGT net scores and SE5 in the SA group.

We used the score of the fifth subjective experience (SE5) as another index of explicit understanding. No significant differences were found in SE5 among the three groups (*p* > 0.05; Table 5). The correlation analysis showed that SE5 was positively correlated with IGT net scores in all subjects (*r* = 0.19, *p* < 0.05; Figure 3). In addition, group analysis showed that there was a significant correlation in the SA group between SE5 and IGT net scores (*r* = 0.70, *p* < 0.05; Figure 4), but no such correlation was found in the other two groups.

**Table 5:** The score of SE5 in three groups.

	***N***	***M***	***SD***	***F***	***p***
SA group	13	3.78	3.49	1.24	0.30
SI group	23	4.65	3.64		
HC group	19	5.68	3.13		

### Psychophysiological Measure

**Figure 5:**
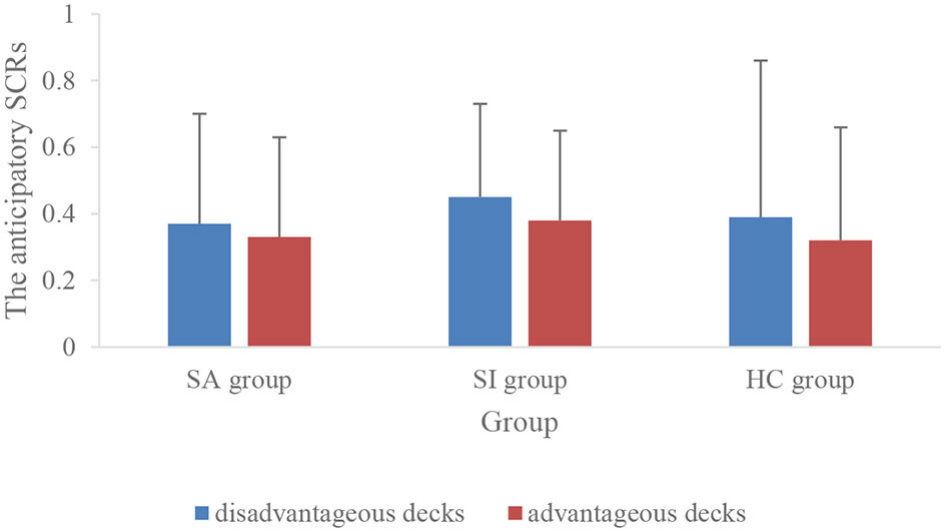
The anticipatory SCRs of disadvantageous and advantageous decks in three groups.

The result of 3 (group) × 2 (type) repeated measurement ANOVA indicated that the main effect of card type was significant (*F* = 15.10, *p* < 0.01, $\eta_{p}^{2}$ = 0.23), i.e., the anticipatory SCRs of the disadvantageous decks were significantly higher than that of advantageous decks (Figure 5). However, the main effect of the group was not significant (*p* > 0.05), and the interaction was not found (*p* > 0.05). The main effect of card type was no more significant after including the age as a covariate in the analysis (*p* > 0.05).

**Figure 6:**
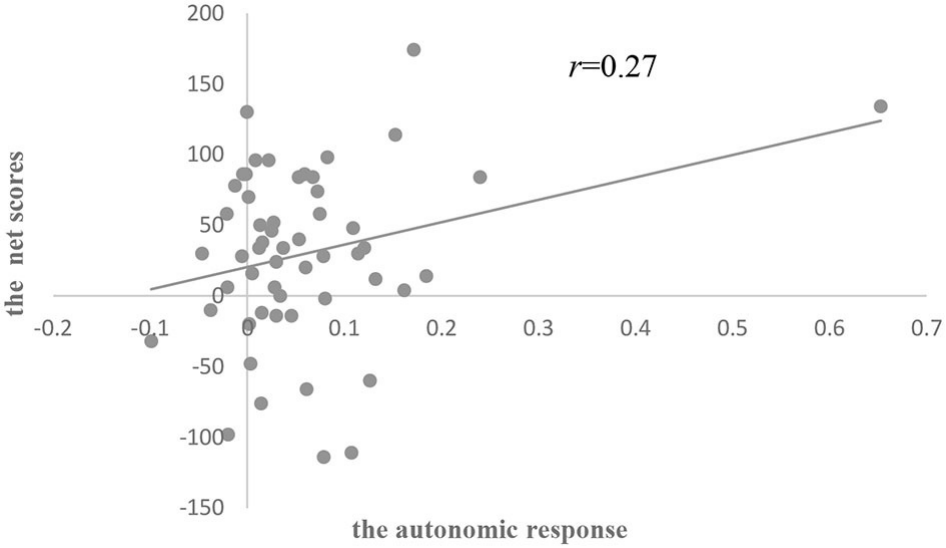
Scatterplot of the relationship between IGT net scores and the autonomic response among all participants.

**Figure 7:**
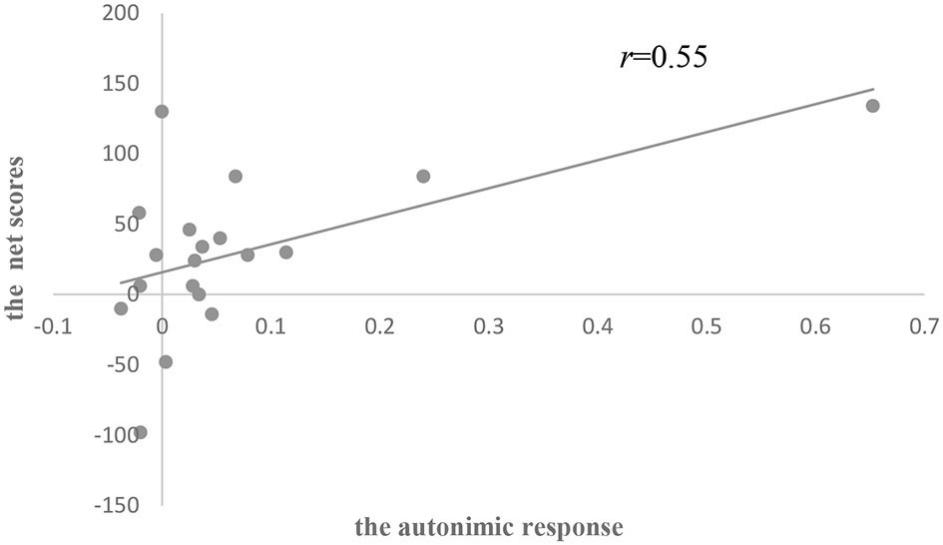
Scatterplot of the relationship between IGT net scores and the autonomic response in the HC group.

The results of ANOVA showed that there was no significant difference in the autonomic response among the three groups (see Table 6). There was a significant positive correlation between the autonomic response and IGT net scores in all subjects (*r* = 0.27, *p* < 0.05; Figure 6). Group analysis showed that there were significant positive correlations between IGT net scores and the autonomic response (*r* = 0.55, *p* < 0.05; Figure 7), and between the anticipatory SCRs of disadvantageous decks and IGT net scores (*r* = 0.45, *p* = 0.05) in the HC group.

**Table 6:** The anticipatory SCRs of disadvantageous and advantageous decks and the autonomic response in three groups.

	**Disadvantageous decks**		**Advantageous decks**		**Autonomic response**		***F***	***p***
	***M***	***SD***	***M***	***SD***	***M***	***SD***		
SA group	0.37	0.33	0.33	0.30	0.04	0.08	0.30	0.74
SI group	0.45	0.28	0.38	0.27	0.06	0.05		
HC group	0.39	0.47	0.32	0.34	0.07	0.15		

### The Relationship of Explicit and Implicit Systems

There were no significant correlations between the SE5 and SCRs, such as, autonomic response (*p* > 0.05), anticipatory SCRs for the disadvantageous decks (*p* > 0.05), and the advantageous decks (*p* > 0.05).

### Relationships Between Decision-Making Performance and Clinical and Personality Variables

There were no significant correlations between IGT net scores and the score of depression (*p* > 0.05), suicide ideation (*p* > 0.05), state anxiety (*p* > 0.05), trait anxiety (*p* > 0.05), and six dimensions of emotional dysregulation (*p* > 0.05).

## Discussion

To our knowledge, this is the first study to explore the influence of explicit knowledge and somatic markers on the decision-making performance of suicidal subjects. No significant differences were found among the three groups on IGT performance, explicit knowledge, and anticipatory SCRs. IGT performance was positively correlated with an index of explicit knowledge (SE5) in suicide attempters and all the subjects, while it was positively correlated with the index of anticipatory SCRs (the difference between the disadvantageous and advantageous decks) in healthy controls and all the subjects.

Behavior results showed that there was no significant difference among the three groups in IGT net scores, and the result was still the same after controlling for age, which was contrary to Hypothesis 1. This result was consistent with the findings of Gorlyn et al. (2013) and Deisenhammer et al. (2018). Gorlyn et al. (2013) found no significant difference between the suicide attempters with depression and control subjects, but violent suicide attempters performed worse in the IGT. Moreover, IGT net scores did not differ significantly among currently depressed suicide attempters, depressed in-patients without suicide behaviors, and healthy controls (Deisenhammer et al., 2018). However, more studies revealed that suicide attempters with mood disorders performed worse than affective and healthy control groups in decision-making tasks (Westheide et al., 2008; Malloy-Diniz et al., 2009; Jollant et al., 2010, 2013; Martino et al., 2011; Bridge et al., 2012). The possible explanations for the inconsistent results could be different sample sources and definitions of suicide attempts (Jollant et al., 2005; Gilbert et al., 2011). Most previous studies were conducted in clinical samples with mood disorders and other psychiatric diagnoses. However, the subjects of our study were recruited from a college students sample and only five subjects in the SA group had psychiatric diagnoses, which may result in a lower pathological level of the subjects. Decision-making deficit was also shown to be associated with major psychiatric disorders, such as normothymic bipolar disorder and depression (Jollant et al., 2007; Caceda et al., 2014). In most previous work, suicide attempts were defined as actual self-injury acts with some intent to die (Jollant et al., 2005, 2010; Gilbert et al., 2011; Bridge et al., 2012). For example, in the study of Jollant et al. (2005), the definition of suicide attempts indicated that patients who exhibited only suicidal ideation or who threatened to commit suicide without actually taking action were not included. However, suicide attempts in our study, according to C-CASA, were defined as potential self-injurious behaviors with certain death intentions, which incorporated aborted (*n*/N = 5/13) and interrupted (*n*/N = 1/13). Moreover, decision-making deficit was found in suicide attempters with violent means rather than non-violent means (Jollant et al., 2005; Gorlyn et al., 2013). Suicide methods of the SA group in this study were all non-violent, such as drug overdose, wrist cutting, and so on, which may contribute to the non-significant result on decision-making performance between suicide attempters and other subjects.

Compared with the HC group, the decision-making performance of suicide ideators was not impaired, which was consistent with Hypothesis 2. There was no significant correlation between suicide ideation and decision-making performance in suicide ideators and all the subjects. There were only three previous studies that explored the decision-making performance of suicide ideators. Moreover, our findings were compatible with the results of Bridge et al. (2012) and Sheftall et al. (2015). For example, Sheftall et al. (2015) did not find group differences between the youths who had suicide ideation in the past 6 months and comparison subjects. However, the study from Westheide et al. (2008) found suicide attempters with current suicide ideation showed impaired decision-making, and suicide ideation was significantly associated with decision-making performance. Therefore, we still cannot conclude that whether there was a decision-making deficit in suicide ideators because of the small number of relative studies and the inconsistent results, which should be resolved in future studies.

In our study, conscious knowledge was indexed by subjective experience and general conscious knowledge. The score of subjective experience was gradually improved in all subjects. This result was replicated with previous studies, which showed that the level of explicit knowledge improved gradually as the decision-making task proceeded in the normal sample (Guillaume et al., 2009; Fernie and Tunney, 2013). There were no significant differences in both measures of explicit knowledge among the three groups. This appeared to show that the explicit system of suicide subjects was not damaged. The study by Jollant et al. (2013) is the first and the only previous study to explore the relationship of the explicit system and decision-making performance in suicide attempters with mood disorders. Their results showed no difference in the level of an explicit understanding of the IGT between suicide attempters with mood disorders and affective controls. It was compatible with our result, which showed that there was no difference in the explicit understanding level between healthy college controls and college suicide attempters with high cognitive levels. The electrophysiological results showed that anticipatory SCRs for disadvantageous decks (A and B) exceeded advantageous decks (C and D) in all subjects. This was in agreement with previous studies in healthy subjects (Wagar and Dixon, 2006; Guillaume et al., 2009; Mardaga and Hansenne, 2012; Yen et al., 2012). Disadvantageous decks induced greater anticipatory SCRs to help participants away from the unfavorable choices (Bechara et al., 1994; Bechara and Damasio, 2005; Sarchiapone et al., 2018). Unfortunately, this result was absent after the control of age. We did not find a significant difference among the three groups in the autonomic response, which might suggest no implicit system impairment in suicide attempters and suicide ideators. In addition, so far there was no previous study that explored whether there is somatic markers deficit in suicidal subjects.

Our correlation results showed that decision-making performance in all the subjects was correlated with explicit knowledge and somatic markers, and there was no correlation between them. Decision-making, therefore, seemed to be associated with the explicit and implicit systems, which is proved by studies conducted in healthy controls (Guillaume et al., 2009; Fernie and Tunney, 2013) and amnesic patients (Gutbrod et al., 2006). Furthermore, IGT net scores were associated with an index of explicit knowledge in suicide attempters and were correlated with the autonomic response of anticipatory SCRs in healthy controls, which implied that distinct strategies were applied in two groups to maintain a similar level of decision-making performance.

The correlations between decision-making performance and somatic markers in healthy controls were consistent with previous research (Carter and Pasqualini, 2004; Wagar and Dixon, 2006; Guillaume et al., 2009; Miu et al., 2012). Success on the IGT was positively correlated with the anticipatory SCRs within a healthy population (Carter and Pasqualini, 2004). Similar SCRs studies also found that overall anticipatory SCRs positively predicted IGT performance of healthy subjects (Wagar and Dixon, 2006; Guillaume et al., 2009; Mardaga and Hansenne, 2012). A meta-analysis by Simonovic et al. (2019) revealed a small-to-medium significant relationship between anticipatory SCRs and IGT performance, which supported the somatic marker hypothesis. Several EEG studies in healthy controls have demonstrated that there was a more negative potential (i.e., Decision Preceding Negativity, DPN) for disadvantageous deck anticipation in the right frontal region (Bianchin and Angrilli, 2011; Giustiniani et al., 2015). Moreover, some functional MRI (fMRI) studies in normal subjects also showed that IGT performance was positively correlated with the activation difference of selecting the unfavorable and the favorable in the ventral media prefrontal lobe (VMPFC, BA10; Fukui et al., 2005) and the left orbital frontal cortex (OFC, BA47; Lawrence et al., 2009). The VMPFC, especially including the OFC region, played a critical role in the process and encoded the outcome-value associations (Rangel et al., 2008; Poppa and Bechara, 2018), which are both in the somatic marker neural circuitry (Li et al., 2010a). However, disrupted VMPFC and OFC value encoding in people with suicide behaviors had been confirmed (Richard-Devantoy et al., 2014; Dombrovski and Hallquist, 2017). Jollant et al. (2010) discovered decreased activation in OFC during risky vs. safe choices in suicide attempters when performing the IGT. Although no impairment of implicit system was found in suicidal subjects in our study, these biological findings could provide some evidence for the potential generation abnormalities of somatic markers in suicide attempters.

Jollant et al. (2013) noted that more explicit knowledge was linked to better IGT performance in healthy and affective controls, but not in suicide attempters. There was no significant IGT performance difference between those who reached or not reached an explicit understanding of suicide attempters. Suicide attempters showed a disconnection between what they know and what they do, and they had deficient use of explicit understanding with the possible impaired implicit system. Therefore, they speculated that the sufficient use of explicit knowledge may be insured when there is an efficient implicit system (Jollant et al., 2013). However, in another study, most post-graduate students had enough knowledge to guide IGT performance after 40 trials and no anticipatory SCRs difference between the bad and good decks in the period before acquiring the knowledge in the normal sample (Fernie and Tunney, 2013). This finding was inconsistent with Jollant et al. (2013) speculation, and the anticipatory SCRs did not show necessary to succeed in the IGT (Gutbrod et al., 2006). Therefore, our results seemed to indicate that, due to the possible difficulty of utilizing somatic markers, the college suicide attempters with high cognition depended more on their explicit knowledge and applied this compensatory strategy to decide as normally as healthy controls.

The limitations of this study should be noted. First, our sample of 36 suicidal individuals could be considered relatively small. It was ambiguous that the performance of suicide attempters in the IGT was on account of the suicide behaviors or the psychiatric symptoms. These results need to be further validated. Second, the influence process of the implicit and explicit systems on decision-making is still in dispute, and the somatic marker hypothesis has been questioned (Fernie and Tunney, 2013; Dong et al., 2016). More factors about the influence on the decision-making process should be discussed in the future. Finally, heart rate, event-related potentials, and other neuroimaging techniques with various strengths could measure the somatic state, which could provide more evidence to the neurophysiological mechanisms during decision-making (Xu and Huang, 2020).

In summary, this study sheds light on the different roles of somatic markers and explicit knowledge on the decision-making performance of healthy controls and suicide attempters. Decision-making in healthy controls was mainly affected by the somatic markers. While the suicide attempters seemed to apply a compensatory strategy by mostly utilizing explicit knowledge to perform as normally as healthy controls in the IGT.

## Data Availability Statement

The original contributions presented in the study are included in the article/supplementary material, further inquiries can be directed to the corresponding author/s.

## Ethics Statement

The studies involving human participants were reviewed and approved by the Ethics Committee of Tianjin University. The patients/participants provided their written informed consent to participate in this study.

## Author Contributions

LLW and JML wrote the manuscript and finished the statistical analysis. JML, HLL, and ZPW collected the data and made a preliminary data analysis. LY and LA contributed to the conception and design of the study. LA revised the manuscript. All authors contributed to the article and approved the submitted version.

## Conflict of Interest

The authors declare that the research was conducted in the absence of any commercial or financial relationships that could be construed as a potential conflict of interest.

## Publisher's Note

All claims expressed in this article are solely those of the authors and do not necessarily represent those of their affiliated organizations, or those of the publisher, the editors and the reviewers. Any product that may be evaluated in this article, or claim that may be made by its manufacturer, is not guaranteed or endorsed by the publisher.

## References

[B1] Ackerman J. P. McBee-Strayer S. M. Mendoza K. Stevens J. Sheftall A. H.. (2015). Risk-sensitive decision-making deficit in adolescent suicide attempters. J. Child Adolesc. Psychopharmacol. 25, 109–113. 10.1089/cap.2014.0041 25265242 PMC4367520

[B2] Bechara A. Damasio A. R. (2005). The somatic marker hypothesis: a neural theory of economic decision. Games Econ. Behav. 52, 336–372. 10.1016/j.geb.2004.06.010

[B3] Bechara A. Damasio A. R. Damasio H. Anderson S. W. (1994). Insensitivity to future consequences following damage to human prefrontal cortex. Cognition 50:3. 10.1016/0010-0277(94)90018-3 8039375

[B4] Bechara A. Damasio H. Damasio A. R. Lee G. P. (1999). Different contributions of the human amygdala and ventromedial prefrontal cortex to decision-making. J. Neurosci. 19, 5473–5481. 10.1523/JNEUROSCI.19-13-05473.1999 10377356 PMC6782338

[B5] Bechara A. Damasio H. Tranel D. Damasio A. R. (1997). Deciding advantageously before knowing the advantageous strategy. Science 275, 1293–1295. 10.1126/science.275.5304.1293 9036851

[B6] Bianchin M. Angrilli A. (2011). Decision preceding negativity in the iowa gambling task: an ERP study. Brain Cogn. 75, 273–280. 10.1016/j.bandc.2011.01.005 21306813

[B7] Bowman C. H. Evans C. E. Y. Turnbull O. H. (2005). Artificial time constraints on the Iowa Gambling Task: the effects on behavioural performance and subjective experience. Brain Cogn. 57, 21–25. 10.1016/j.bandc.2004.08.015 15629209

[B8] Bridge J. A. McBee-Strayer S. M. Cannon E. A. Sheftall A. H. Reynolds B. (2012). Impaired decision making in adolescent suicide attempters. J. Am. Acad. Child Adolesc. Psychiatry 51, 394–403. 10.1016/j.jaac.2012.01.002 22449645 PMC3314230

[B9] Caceda R. Nemeroff C. B. Harvey P. D. (2014). Toward an understanding of decision making in severe mental illness. J. Neuropsychiatr. Clin. Neurosci. 26, 196–213. 10.1176/appi.neuropsych.12110268 24599051

[B10] Carter S. Pasqualini M. S. (2004). Stronger autonomic response accompanies better learning: a test of Damasio's somatic marker hypothesis. Cogn. Emot. 18, 901–911. 10.1080/02699930341000338

[B11] Chamberlain S. R. Odlaug B. L. Schreiber L. R. N. Grant J. E. (2013). Clinical and neurocognitive markers of suicidality in young adults. J. Psychiatric Res. 47, 586–591. 10.1016/j.jpsychires.2012.12.016 23357208

[B12] Clark L. Dombrovski A. Y. Siegle G. J. Butters M. A. Shollenberger C. L. (2011). Impairment in risk-sensitive decision-making in older suicide attempters with depression. Psychol. Aging 26, 321–330. 10.1037/a0021646 21443349 PMC3115442

[B13] Deisenhammer E. A. Schmid S. K. Kemmler G. Moser B. Delazer M. (2018). Decision making under risk and under ambiguity in depressed suicide attempters, depressed non-attempters and healthy controls. J. Affect. Disord. 226, 261–266. 10.1016/j.jad.2017.10.012 29020650

[B14] Desmyter S. van Heeringen C. Audenaert K. (2011). Structural and functional neuroimaging studies of the suicidal brain. Progr. Neuro Psychopharmacol. Biol. Psychiatr. 35, 796–808. 10.1016/j.pnpbp.2010.12.026 21216267

[B15] Dombrovski A. Y. Clark L. Siegle G. J. Butters M. A. Ichikawa N. (2010). Reward/Punishment reversal learning in older suicide attempters. Am. J. Psychiatr. 167, 699–707. 10.1176/appi.ajp.2009.09030407 20231320 PMC3020386

[B16] Dombrovski A. Y. Hallquist M. N. (2017). The decision neuroscience perspective on suicidal behavior. Curr. Opin. Psychiatr. 30, 7–14. 10.1097/YCO.0000000000000297 27875379 PMC5291285

[B17] Dong X. Du X. Qi B. (2016). Conceptual knowledge influences decision making differently in individuals with high or low cognitive flexibility: an ERP study. PLoS ONE 11:e158875. 10.1371/journal.pone.0158875 27479484 PMC4968815

[B18] Falcone T. Staniskyte M. Forcen F. E. Vengoechea J. (2018). Neurobiology of suicide. Suicide Prev. 2018, 3–21. 10.1007/978-3-319-74391-2_1

[B19] Fernie G. Tunney R. J. (2013). Learning on the IGT follows emergence of knowledge but not differential somatic activity. Front. Psychol. 4:687. 10.3389/fpsyg.2013.00687 24109462 PMC3790076

[B20] Fukui H. Murai T. Fukuyama H. Hayashi T. Hanakawa T. (2005). Functional activity related to risk anticipation during performance of the Iowa gambling task. NeuroImage 24, 253–259. 10.1016/j.neuroimage.2004.08.028 15588617

[B21] Gilbert A. M. Garno J. L. Braga R. J. Shaya Y. Goldberg T. E. (2011). Clinical and cognitive correlates of suicide attempts in bipolar disorder. J. Clin. Psychiatr. 72, 1027–1033. 10.4088/JCP.10m06410 21813075 PMC4035109

[B22] Giner L. Blasco-Fontecilla H. De La Vega D. Courtet P. (2016). Cognitive, emotional, temperament, and personality trait correlates of suicidal behavior. Curr. Psychiatr. Rep. 18:742. 10.1007/s11920-016-0742-x 27726066

[B23] Giustiniani J. Gabriel D. Nicolier M. Monnin J. Haffen E. (2015). Neural correlates of successful and unsuccessful strategical mechanisms involved in uncertain Decision-Making. PLoS ONE 10:e130871. 10.1371/journal.pone.0130871 26086196 PMC4472228

[B24] Gorlyn M. Keilp J. G. Oquendo M. A. Burke A. K. John Mann J. (2013). Iowa Gambling Task performance in currently depressed suicide attempters. Psychiatr. Res. 207, 150–157. 10.1016/j.psychres.2013.01.030 23489594

[B25] Guillaume S. Jollant F. Jaussent I. Lawrence N. Malafosse A. (2009). Somatic markers and explicit knowledge are both involved in decision-making. Neuropsychologia 47, 2120–2124. 10.1016/j.neuropsychologia.2009.04.003 19427005

[B26] Gutbrod K. Krouzel C. Hofer H. Muri R. Perrig W. (2006). Decision-making in amnesia: do advantageous decisions require conscious knowledge of previous behavioural choices? Neuropsychologia 44, 1315–1324. 10.1016/j.neuropsychologia.2006.01.014 16513144

[B27] Jollant F. Bellivier F. Leboyer M. Astruc B. (2005). Impaired decision making in suicide attempters. Am. J. Psychiatr. 162, 304–310. 10.1176/appi.ajp.162.2.304 15677595

[B28] Jollant F. Guillaume S. Jaussent I. Bechara A. Courtet P. (2013). When knowing what to do is not sufficient to make good decisions: deficient use of explicit understanding in remitted patients with histories of suicidal acts. Psychiatr. Res. 210, 485–490. 10.1016/j.psychres.2013.07.011 23972765

[B29] Jollant F. Guillaume S. Jaussent I. Bellivier F. Leboyer M. (2007). Psychiatric diagnoses and personality traits associated with disadvantageous decision-making. Eur. Psychiatr. 22, 455–461. 10.1016/j.eurpsy.2007.06.001 17764910

[B30] Jollant F. Lawrence N. L. Olié E. Guillaume S. Courtet P. (2011). The suicidal mind and brain: a review of neuropsychological and neuroimaging studies. World J. Biol. Psychiatr. 12, 319–339. 10.3109/15622975.2011.556200 21385016

[B31] Jollant F. Lawrence N. S. Olie E. O'Daly O. Malafosse A. (2010). Decreased activation of lateral orbitofrontal cortex during risky choices under uncertainty is associated with disadvantageous decision-making and suicidal behavior. NeuroImage 51, 1275–1281. 10.1016/j.neuroimage.2010.03.027 20302946

[B32] Lawrence N. S. Jollant F. O'Daly O. Zelaya F. Phillips M. L. (2009). Distinct roles of prefrontal cortical subregions in the Iowa Gambling Task. Cerebral Cortex 19, 1134–1143. 10.1093/cercor/bhn154 18787233

[B33] Li X. Lu Z. D'Argembeau A. Ng M. Bechara A. (2010a). The iowa gambling task in fMRI images. Hum Brain Map. 31, 410–423. 10.1002/hbm.20875 19777556 PMC2826566

[B34] Li X. Phillips M. R. Tong Y. Li K. Zhang Y. Zhang Y.. (2010b). Reliability and validity of the Chinese version of Beck Suicide Ideation Scale (BSI-CV) in adult community residents. Chin. Mental Health J. 24, 250–255. 10.3969/j.issn.1000-6729.2010.04.003

[B35] Maia T. V. McClelland J. L. (2004). A reexamination of the evidence for the somatic marker hypothesis: what participants really know in the Iowa gambling task. Proc. Natl. Acad. Sci. U. S. A. 101, 16075–16080. 10.1073/pnas.0406666101 15501919 PMC528759

[B36] Malloy-Diniz L. F. Neves F. S. Abrantes S. S. C. Fuentes D. Corrêa H. (2009). Suicide behavior and neuropsychological assessment of type I bipolar patients. J. Affect. Disord. 112, 231–236. 10.1016/j.jad.2008.03.019 18485487

[B37] Mann J. J. Rizk M. M. (2020). A Brain-Centric model of suicidal behavior. Am. J. Psychiatr. 177, 902–916. 10.1176/appi.ajp.2020.20081224 32998550 PMC7676389

[B38] Mann J. J. Waternaux C. Haas G. L. Malone K. M. (1999). Toward a clinical model of suicidal behavior in psychiatric patients. Am. J. Psychiatr. 156, 181–189. 9989552 10.1176/ajp.156.2.181

[B39] Mardaga S. Hansenne M. (2012). Personality and skin conductance responses to reward and punishment influence on the iowa gambling task performance. J. Individ. Differ. 33, 17–23. 10.1027/1614-0001/a000057

[B40] Martino D. J. Strejilevich S. A. Torralva T. Manes F. (2011). Decision making in euthymic bipolar I and bipolar II disorders. Psychol. Med. 41, 1319–1327. 10.1017/S0033291710001832 20860871

[B41] Miu A. C. Crişan L. G. Chiş A. Ungureanu L. Drugă B. Vulturar R. (2012). Somatic markers mediate the effect of serotonin transporter gene polymorphisms on Iowa Gambling Task. Genes Brain Behav. 11, 398–403. 10.1111/j.1601-183X.2012.00774.x 22348680

[B42] Overman W. H. Pierce A. (2013). Iowa Gambling Task with non-clinical participants: effects of using real + virtual cards and additional trials. Front. Psychol. 4:935. 10.3389/fpsyg.2013.00935 24376431 PMC3859904

[B43] Perrain R. Dardennes R. Jollant F. (2021). Risky decision-making in suicide attempters, and the choice of a violent suicidal means: an updated meta-analysis. J. Affect. Disord. 280, 241–249. 10.1016/j.jad.2020.11.052 33220560

[B44] Poppa T. Bechara A. (2018). The somatic marker hypothesis: revisiting the role of the 'body-loop' in decision-making. Curr. Opin. Behav. Sci. 19, 61–66. 10.1016/j.cobeha.2017.10.007

[B45] Posner K. Brown G. K. Stanley B. Brent D. A. Yershova K. V. Oquendo M. A.. (2011). The Columbia-Suicide Severity Rating Scale: initial validity and internal consistency findings from three multisite studies with adolescents and adults. Am. J. Psychiatr. 168, 1266–1277. 10.1176/appi.ajp.2011.10111704 22193671 PMC3893686

[B46] Posner K. Oquendo M. A. Gould M. Stanley B. Davies M. (2007). Columbia Classification Algorithm of Suicide Assessment (C-CASA): classification of suicidal events in the FDA's pediatric suicidal risk analysis of antidepressants. Am. J. Psychiatr. 164, 1035–1043. 10.1176/ajp.2007.164.7.1035 17606655 PMC3804920

[B47] Rangel A. Camerer C. Montague P. R. (2008). A framework for studying the neurobiology of value-based decision making. Nat. Rev. Neurosci. 9, 545–556. 10.1038/nrn2357 18545266 PMC4332708

[B48] Richard-Devantoy S. Berlim M. T. Jollant F. (2014). A meta-analysis of neuropsychological markers of vulnerability to suicidal behavior in mood disorders. Psychol. Med. 44, 1663–1673. 10.1017/S0033291713002304 24016405

[B49] Richard-Devantoy S. Olié E. Guillaume S. Bechara A. Courtet P. (2013). Distinct alterations in value-based decision-making and cognitive control in suicide attempters: toward a dual neurocognitive model. J. Affect. Disord. 151, 1120–1124. 10.1016/j.jad.2013.06.052 23876195

[B50] Sarchiapone M. Gramaglia C. Iosue M. Carli V. Mandelli L. (2018). The association between electrodermal activity (EDA), depression and suicidal behaviour: a systematic review and narrative synthesis. BMC Psychiatr. 18:22. 10.1186/s12888-017-1551-4 29370787 PMC5785904

[B51] Sheftall A. H. Davidson D. J. McBee-Strayer S. M. Ackerman J. Mendoza K. (2015). Decision-making in adolescents with suicidal ideation: a case-control study. Psychiatry Res. 228, 928–931. 10.1016/j.psychres.2015.05.077 26163723 PMC6207944

[B52] Simonovic B. Stupple E. Gale M. Sheffield D. (2019). Sweating the small stuff: a meta-analysis of skin conductance on the Iowa gambling task. Cogn. Affect. Behav. Neurosci. 19, 1097–1112. 10.3758/s13415-019-00744-w 31493212 PMC6785590

[B53] van Heeringen K. Mann J. J. (2014). The neurobiology of suicide. Lancet Psychiatr. 1, 63–72. 10.1016/S2215-0366(14)70220-2 26360403

[B54] Wagar B. M. Dixon M. (2006). Affective guidance in the Iowa gambling task. Cogn. Affect. Behav. Neurosci. 6, 277–290. 10.3758/CABN.6.4.277 17458443

[B55] Wang L. Liu H. Li Z Du W. (2007). A Study on the reliability and validity of the Chinese version of the Emotional regulation Questionnaire. Chin. Health Psychol. J. 6, 503–505. 10.3969/j.issn.1005-1252.2007.06.034

[B56] Wang X. D. (1999). The Handbook of Mental Health Rating Scale [in Chinese]. Peking: Chinese Mental Health Journal Press.

[B57] Westheide J. Quednow B. B. Kuhn K. Hoppe C. Cooper-Mahkorn D. (2008). Executive performance of depressed suicide attempters: the role of suicidal ideation. Eur. Archiv. Psychiatr. Clin. Neurosci. 258, 414–421. 10.1007/s00406-008-0811-1 18330667

[B58] World Health Organization (2017). Depression and Other Common Mental Disorders: Global Health Estimates. Geneva: World Health Organization.

[B59] Xu F. Huang L. (2020). Electrophysiological measurement of emotion and somatic state affecting ambiguity decision: evidences from SCRs, ERPs, and HR. Front. Psychol. 11:899. 10.3389/fpsyg.2020.00899 32477219 PMC7240102

[B60] Yen N. Chou I. Chung H. Chen K. (2012). The interaction between expected values and risk levels in a modified Iowa gambling task. Biol. Psychol. 91, 232–237. 10.1016/j.biopsycho.2012.06.008 22820038

